# Carotid Disease and Ageing: A Literature Review on the Pathogenesis of Vascular Senescence in Older Subjects

**DOI:** 10.1155/2020/8601762

**Published:** 2020-06-13

**Authors:** Eleni Sertedaki, Dimitris Veroutis, Flora Zagouri, George Galyfos, Konstadinos Filis, Alexandros Papalambros, Konstantina Aggeli, Panagiota Tsioli, George Charalambous, George Zografos, Fragiska Sigala

**Affiliations:** ^1^First Department of Propaedeutic Surgery, Hippocration General Hospital, School of Medicine, National Kapodistrian University of Athens, Athens, Greece; ^2^Molecular Carcinogenesis Group, Department of Histology and Embryology, School of Medicine, National Kapodistrian University of Athens, Athens, Greece; ^3^Clinical Therapeutics Department, Alexandra General Hospital, School of Medicine, National Kapodistrian University of Athens, Athens, Greece; ^4^First Department of Surgery, Laikon General Hospital, School of Medicine, National Kapodistrian University of Athens, Athens, Greece; ^5^First Department of Cardiology, University of Athens Medical School, Hippocration Hospital, Athens, Greece; ^6^First Department of Pathology, Laikon General Hospital, School of Medicine, National Kapodistrian University of Athens, Athens, Greece

## Abstract

Aging is a natural process that affects all systems of the human organism, leading to its inability to adapt to environmental changes. Advancing age has been correlated with various pathological conditions, especially cardiovascular and cerebrovascular diseases. Carotid artery (CA) is mainly affected by age-induced functional and morphological alterations causing atheromatous disease. The evolvement of biomedical sciences has allowed the elucidation of many aspects of this condition. Symptomatic carotid disease (CD) derives from critical luminar stenosis or eruption of an atheromatous plaque due to structural modifications of the vessels, such as carotid intima-media thickening. At a histologic level, the aforementioned changes are mediated by elastin fragmentation, collagen deposition, immune cell infiltration, and accumulation of cytokines and vasoconstrictors. Underlying mechanisms include chronic inflammation and oxidative stress, dysregulation of cellular homeostatic systems, and senescence. Thus, there is an imbalance in components of the vessel wall, which fails to counteract exterior stress stimuli. Consequently, arterial relaxation is impaired and atherosclerotic lesions progress. This is a review of current evidence regarding the relationship of aging with vascular senescence and CD. A deeper understanding of these mechanisms can contribute to the production of efficient prevention methods and targeted therapeutic strategies.

## 1. Introduction

Carotid disease (CD) is one of the most common age-related diseases. It refers to a number of macroscopic morphological changes and to alterations at cytopathological level in common carotid artery (CCA) and internal carotid artery (ICA) that lead to cerebrovascular events such as strokes and transient ischemic attacks (TIAs) that comprise a major cause of mortality and morbidity, especially amongst the elderly [1].

Occurrence of CD is strongly associated with cardiovascular disease, atherosclerosis, and arterial stiffening induced by aging as well as by various atherogenic risk factors, including metabolic syndrome, hypertension, dyslipidemia, especially high low-density lipoprotein (LDL) levels, smoking, and diabetes mellitus (DM). Particularly, aging itself has been identified actually as an independent and unmodifiable risk factor for arterial stiffening and CD irrespective of patient comorbidities, whilst carotid atheromatous disease is considered a typical manifestation of vascular aging or vascular senescence [1, 2].

Vascular senescence refers to the establishment of clinical and subclinical structural and functional changes at a histological and cellular level, involving cells and the extracellular matrix of vessel walls [3] that are related with the loss of its homeostatic potential to adapt to environmental stress stimuli. It is a separate entity from aging, provoked by ageing itself, but also by the aforementioned atherogenic factors [2]. It is also correlated with “inflammaging,” a sterile, low-grade, chronic inflammation induced by advancing age, leading to a number of diseases, mostly cardiovascular and cerebrovascular ones [4].

In fact, aging is a natural process that causes multiple changes to the human organism decreasing its ability to maintain its homeostasis and make it more vulnerable to threats. It is associated with the impairment of vascular function and onset of cardiovascular disease [5, 6] even in the absence of other risk factors [7, 8]. There seems to be a linear relationship between arterial stiffness, age [9], and accelerated arterial stiffening between 50 and 60 years of age [10].

Multiple macrovascular and microvascular complications are initiated due to the formation of atheromatous lesions, under the impact of oxidized LDLs (oxLDLs), endothelial dysfunction, and impaired arterial wall homeostasis, which in turn exacerbate media thickening and fibrosis, leading to arterial stiffness and cerebrovascular disease [5, 6, 11–19]. As atherosclerosis is a disease of aging that involves cellular senescence, a deep understanding of the underlying mechanisms that regulate cell survival and onset of atheromatous disease with increasing age is a valuable tool in prevention and management of the progression of CD [4, 6, 10].

The purpose of this review is to summarize existing findings regarding aging and progression of carotid atheromatous disease. Thus, we have collected data through a comprehensive literature search online. We believe that deciphering molecular mechanisms involved in the pathogenesis of the disease will give us new perspectives in the prevention, diagnosis, and treatment of vascular disease in older ages.

### 1.1. Macroscopic Changes in Carotid Artery with Advancing Age

Anatomical and geometrical features of the carotid artery (CA) play an important role in the onset of ICA stenosis and occurrence of CD, manifested as transient ischemic attack, stroke, or syncope [8, 20]. Such morphological changes take place with advancing age, including increases in vessel volume, diameter, and rotation of the CCA and ICA, in the bifurcation and ICA angles. A higher anatomical position of ICA has also been detected in patients over 60 years old. Moreover,, the ICA angle seems to affect ICA radius and degree of stenosis, while the bifurcation angle seems to influence carotid circulation, constituting one of the most common sites for the formation of atherosclerotic plaques [20]. The aforementioned changes alter the hemodynamic forces that affect crucially the progression of atherosclerosis.

However, prior to the establishment of these morphological changes, functional deterioration of vascular wall takes place. Arterial stiffness is known to progress with age and elastic properties of CA which tend to differentiate [21]. Vascular functionality is reduced significantly by arterial stiffness, as the vessel wall fails to adapt to pressure fluctuations [8]. According to ultrasound findings, the carotid *β*-stiffness index (ultrasound parameter-assessing arterial stiffening) and vessel diameter increase, while arterial strain (ability to adjust with pressure changes with mechanical deformation) reduces with advancing age [22]. Consistently, carotid intima-media thickness (cIMT), a common measure of atherosclerosis and useful tool for the prediction of cardiovascular and cerebrovascular events, displays a linear increase along with age irrespectively of other factors [23, 24]. Furthermore, complicated carotid atheromas with intraplaque hemorrhage are related with older age as confirmed in multiple studies with use of ultrasound, magnetic resonance, and histological assessment of carotid plaques [25, 26].

At a histologic level, it is known that the hallmark of atherosclerosis is the formation of an atheromatous lesion. Hemodynamic shear stress results in endothelial injury and expression of cell adhesion molecules (CAMs) and other cell surface receptors that lead to increased endothelial permeability and accumulation of vascular smooth muscle cells (VSMCs) in the subendothelial space. Circulating LDL molecules, especially when at high serum concentrations, enter into subendothelial spaces, bind to glycoproteins, and are oxidized by free endothelial cell receptors. OxLDLs promote transendothelial migration of monocytes into the subendothelial spaces and their differentiation to macrophages under the impact of cytokines. These macrophages absorb oxLDLs becoming foam cells that stimulate a local inflammatory reaction, which further attracts inflammatory cells initiating lesion formation. This atheromatous lesion is made up of a lipid core and a fibrous cap consisting of macrophages, VSMCs, collagen, elastin, and proteoglycans [27].

Stable plaques that are at a solid state have a thick fibrous cap, while vulnerable or unstable ones are at a rather liquid state having a large lipid core and a thin fibrous cap related with inflammatory infiltrates [25–27].

Evidently, the progression of atheromatous plaques at a histologic level seems to be enhanced by advancing age, as their morphology is altered towards instability and vulnerability to rupture. Plaques become more atheromatous, inflammatory, and less fibrous, with a decrease in elastin fibers, VSMCs, and overall cellularity. Lipid accumulation, calcification, hemorrhage, and deficient tissue repair that make plaques more prone to injury are identified. Macrophage infiltrates are also detected mainly in plaques of symptomatic old patients rather than asymptomatic ones [26]. Moreover, plaques from elderly patients contain less fractalkine, interferon-gamma (IFN-*γ*), and tumor necrosis factor-*α* (TNF-*α*), a finding suggesting that infiltrating macrophages in elderly are probably less functional. They also have a high content of the proinflammatory and prothrombotic molecule sCD40L and vascular endothelial growth factor (VEGF), which is a stimulus for plaque neovascularization at conditions of inflammation and poor blood supply. The concurrent reduction of endothelial shear stress and increase of arterial stiffness and wall tension promote atherosclerosis. Consequently, the age-associated plaque morphology seems to change towards more vulnerable plaques, [25, 28] the rupture of which leads to acute cerebrovascular and cardiovascular events, which increase in incidence with age.

## 2. Underlying Mechanisms

### 2.1. Endothelial Dysfunction

Endothelial cells (ECs) form a thin layer between circulating blood and the rest of the vessel wall. Endothelium is a strategically placed barrier with an active role on vascular homeostasis [29]. In response to physical and chemical signals, ECs produce multiple substances that regulate nutrient supply, cellular adhesion, coagulation, and fibrinolysis, as well as VSMC proliferation, vessel wall inflammation, and vascular tone [30]. In particular, ECs have autocrine, paracrine, and endocrine functions. They produce prothrombotic and antithrombotic substances, as well as both fibrinolytic and antifibrinolytic ones. They regulate VSMC migration to the intima as well as leucocyte adhesion and activation [27, 28]. In addition, the regulation of vascular tone is mediated by endothelium-derived factors (vasodilators and vasoconstrictors) that affect vascular structure and end-organ perfusion in the long term. Especially, nitric oxide (NO) is a critical vasorelaxant factor generated by the endothelial NO synthase (eNOS) [31, 32]. However, when ECs are activated by prolonged stress stimuli, such as aging and atherogenic factors, they become senescent or undergo apoptosis that leads to the loss of inegrity of the endothelial layer. They lose their capacity to maintain homeostasis, and they are invaded by lipids, platelets, and leucocytes that stimulate plaque formation [27, 28].

### 2.2. Endothelial Dysfunction due to Nitric Oxide (NO) Depletion

Endothelial dysfunction refers to the inability of endothelium to maintain vascular homeostasis, enhancing vasoconstriction, oxidative, and inflammatory reactions that lead to various cardiovascular diseases. Impaired vasodilation in hypercholesterolemia results mainly from diminishment of NO bioavailability [33]. Moreover,, many of the aforementioned protective endothelial functions including inhibition of platelet and leucocyte adhesion and aggregation, VSMC migration, and inhibition of LDL clustering are dysregulated due to NO reduced bioavailability, resulting in the evolution of atheromatous disease [27, 28, 31, 32]. According to experimental and clinical data, increasing age is related with reduced levels of the endothelial NO synthase (eNOS), thus reduced bioavailability of NO, and increased levels of the inducible NO synthase (iNOS), which is upregulated under inflammatory conditions producing excessive amounts of NO that rapidly interact with oxidative species forming ONOO- that exacerbates nitrosative stress and deteriorates endothelial homeostasis [16, 27, 33]. This imbalance in synthesis and degradation of NO ends up to its depletion and loss of its beneficial effects. Thus, atheromatous disease evolves further due to the diminished NO generation and impaired NO diffusion from endothelium to VSMCs [16, 27, 33]. Consistently, iNOS inhibition could improve endothelium-dependent responses according to experimental data [33, 34].

### 2.3. Endothelial Dysfunction due to Increased Production of Vasoconstrictors

Another enzyme involved in ageing-induced endothelial dysfunction is cyclooxygenase (COX). It leads to generation of vasoconstrictor substances, such as thromboxane A2 (TXA2) and prostaglandins (PGs), such as PGH2, PGF2*α*, and PGE2, which deteriorate endothelial function [35–37]. In addition, activation of endothelin-1 is reinforced with increasing age, enhancing vasoconstriction and inflammation [38]. Endothelial vasoconstrictors have been shown to cause chemotactic accumulation of leukocytes and platelets mediated by the expression of surface adhesion molecules (selectins, integrins, immunoglobulins, intercellular adhesion molecule-1 (ICAM-1), vascular cell adhesion molecule-1 (VCAM-1), etc.). Thus, monocytes adhere to the endothelial surface and migrate into the intima [27, 28, 38]. COX inhibitors, such as indomethacin, have been shown to conserve vascular functionality in older adults and aged animals [39]. Evidence remains inconsistent, though, as some studies shew no effect with the use of COX-2 inhibitors [40]. Nevertheless, the lack of prostaglandin I2 (PGI2)-mediated vasodilatation with aging has been documented both in vivo and in vitro [41].

### 2.4. Chronic Vascular Inflammation

The major underlying factor is low-grade chronic inflammation detected in aged vasculature. It acts as a continuous stress stimulus that promotes oxidative strain, cellular senescence, and impairment of vascular function [42].

In this context, multiple cytokines are expressed composing networks that get differentiated with increasing age irrespectively of other risk factors [43]. Blood samples from older adults reveal prevalence of C-reactive protein (CRP) as well as of proinflammatory cytokines and adhesion molecules such as tumor necrosis factor-*α* (TNF-*α*), interleukin (IL)-1*β*, interleukin-6 (IL-6), and VCAM-1 [43]. Overexpression of these molecules is associated with aggravation of endothelium impairment and arterial stiffening [44–46] that lead to cerebrovascular and cardiovascular events, and increased mortality and morbidity amongst the elderly [46, 47]. It is worth mentioning that inflammatory mediators, such as TNF-*α* that regulates eNOS and iNOS expression, are produced by ECs too [48, 49].

On the contrary, anti-inflammatory cytokines, such as interleukin-10 (IL-10) and adiponectin, which decelerate senescence and protect arteries against cardiovascular disease, seem to be downregulated with increasing age [50–54]. In particular, inhibition of IL-10, known to protect from carotid atheromatous disease and coronary calcification, [50] led to endothelial dysfunction and vascular inflammation of carotid arteries in IL-10 knockout aged mice according to experimental findings [55, 56].

Moreover, immune cell infiltrates with macrophages and T lymphocytes have been detected in histologic carotid lesions conserving inflammatory phenotype in aged subjects [57, 58]. Consistently, macrophage and leukocyte infiltrates have been detected in aortic adventitia and perivascular fat tissue of aged mice [44, 58, 59].

Nuclear factor kappa-B (NF-*κ*Β), a thoroughly studied protein complex, is considered to have a critical role in the initiation and conservation of inflammation as well as in the induction of senescence [60, 61]. It remains inactive linked to protein I*κ*B-*α* in the cytoplasm; when activated by stress stimuli, NF-*κ*Β is transferred to nucleus, where it controls expression of numerous target genes [60–64] activating TNF-*α*, interleukins (IL-1*β* and IL-6), chemokines (IL-8 and RANTES), adhesion molecules (intercellular adhesion molecule and vascular cell adhesion molecule), and enzymes (iNOS and COX-2) [60, 61, 64, 65]. Through a vicious cycle, NF-*κ*Β expression is reinforced by the products of its target-signaling cascades [61, 64, 65]. Thus, it constitutes a major regulator of immune vascular responses to aging and its inhibition could delay ageing process [66–71].

### 2.5. Oxidative Stress

In addition to chronic inflammation, chronic oxidative stress is a main feature of the aging process [16, 69, 72]. Reactive oxygen species (ROS) (superoxide anion and hydrogen peroxide) generation is mediated by various oxidant enzymes (e.g., NADPH oxidase (NOX), xanthine oxidase, cytochromes, and uncoupled eNOS) that are upregulated with advancing age; mitochondrial hydrogen peroxide is an important source of ROS in aging vasculature too [73–77]. This increase though would stimulate cellular antioxidant defensive mechanisms. However, there is a significant decrease in critical antioxidant enzymes (dismutases, catalases, and deacetylases) under the impact of numerous inflammatory factors, such as NF-*κ*Β [78]. Thus, antioxidant capacity is reduced in the elderly, leading to an imbalance in favor of oxidants on the vascular wall [72, 79]. In support of this, elimination of oxidative stress could improve or reverse aging-induced vascular dysfunction according to findings of multiple experimental studies, constituting a feasible targeted therapeutic approach [78–80].

Furthermore, although native LDL molecules are inactive, oxidative stress involved in atherosclerosis process, turns them into the highly immunogenic oxLDLs, which trigger the production of endothelial adhesion molecules, attract monocytes, and enhance proinflammatory gene activity [16, 27, 74]. In fact, this disequilibrium between pro and antioxidant factors increases endothelial permeability and promotes foam cell formation [73, 74, 81].

## 3. Cell Regulatory Systems

### 3.1. Role of microRNAs

Cell modulatory systems include microRNAs, small noncoding RNAs that bind to 3′ untranslated regions of mRNAs regulating their stability and translation [82]. Due to their ability to interact with multiple mRNAs, miRNAs have a critical role in controlling signaling pathways related with inflammation, senescence, and aging [82–90] miRNAs with inflammatory potential, such as miR-34a (the inducer of vascular smooth muscle senescence and arterial dysfunction), [91] miR-146a (the senescence and inflammation inducer interacting with telomeres and telomerase) [90, 92], and miR-125a-5p (the dysregulator of eNOS and VEGF) are upregulated in vascular cells of older subjects and involved in different signaling pathways (e.g., NF-*κ*B activation) [63, 88]. Conversely, miRNAs with anti-inflammatory effects such as miR-155 and miR-27a are downregulated [93–95].

### 3.2. Other Cell Regulatory Systems

Additional modulatory systems with critical cellular homeostatic functions are involved in vascular inflammation and cellular senescence that evolves with ageing [95–98].

To begin with, SIRT1, a NAD-dependent histone deacetylase, a member of the sirtuins family, plays a crucial role in cell survival and metabolism [97–99]. It is mostly expressed in the nucleus where it regulates transcription of multiple genes. It inhibits NF-*κ*B with a negative impact on senescence [96, 99]. Its levels though are diminished in aged subjects, with an enhancement of ROS production by NADPH oxidases that remain unrestrained. As a result, inflammation, endothelial, and smooth muscle cell dysfunction and arterial stiffness progress [100–102]. Restoration of SIRT1 levels (e.g., with resveratrol) is a promising therapeutic target to reverse vascular inflammation and endothelial dysfunction [101–105].

Likewise, SIRT6, another nuclear deacetylase, has similar effects. It suppresses NF-*κ*B activity, inhibits oxidative stress, ageing phenotype, and senescence, expanding lifespan, according to preclinical data on mouse models and human ECs [106–109]. SIRT6 expression is downregulated by inflammatory stimuli though, including ageing [110, 111].

Another regulatory molecule is nuclear-related factor-2 (Nrf2) that controls cell responses to oxidative stress in ageing process [112–114]. It represses NF-*κ*Β activation and nuclear translocation, [67, 68] while NF-*κ*B antagonizes Nrf2 signaling at the transcription level, according to experimental studies on aorta and carotid arteries from aged animals [70, 72, 114]. Furthermore, Nrf-2 stimulates a cytoprotective kind of angiogenesis through enhancement of angiogenic potential of endothelial cells, which is impaired with advancing age along with the reduction of NRf-2 levels, while inflammation and ischemia-related neovascularization is amplified [16, 115].

Finally, AMPK (AMP-activated kinase) and mTOR (mammalian target of rapamycin) are additional modulatory factors. They are crucial energy sensors with important roles on cell metabolism and survival. Moreover, they have been identified to antagonize each other; AMPK can directly inhibit mTOR while high levels of AMPK and low levels of mTOR are associated with longevity. Hence, AMPK, which affects intracellular pathways critical for cell cycle and metabolism, is downregulated in arteries of old subjects. On the other hand, mTOR signaling is enhanced in aging and related diseases and its inhibition seems to exert antioxidant and protective effects on endothelium [116–119].

### 3.3. Autophagy and Senescence

Autophagy refers to deterioration of nonfunctional proteins and organnelles [44, 120, 121] and apoptosis to the programmed death of damaged cells in order to ensure overall cellularity survival and tissue homeostasis [122, 123]. However, with age, complex changes in the immune system take place and both procedures are found reduced [57, 58]. Accumulating evidence indicates that advancing age entails a dysregulation of all homeostatic systems enhancing inflammatory and oxidative phenotype [60, 65, 124] genomic instability, telomere damage, and stress-related p53/p21 and p16/pRB cellular pathways. In consequence, cellular senescence, a state of long-term cycle arrest that eliminates cellular functionality and regenerative potential, occurs [125–129].

Senescent cells (endothelial and smooth muscle cells) are directly related with pathogenic mechanisms of vascular dysfunction in multiple ways. They display reduced proliferative potential, impaired microangiogenesis, and multiple autocrine and paracrine actions known as SASP (senescence-associated secretory phenotype). The generated substances have proinflammatory and oxidant effects on neighboring cells deteriorating vascular functionality. Consistently, senolytic therapies seem to decelerate aging complications and endothelial dysfunction [130–134].

### 3.4. Extracellular Matrix Changes

All mechanisms, described above to affect intracellular and cellular components of the arterial wall, equally affect the extracellular matrix, which displays characteristic changes with advancing age, such as elastin fragmentation, collagen accumulation, and matrix protein cross-linking. Elastolysis and fibrosis derive from the actions of metalloproteinases (MMP-2 and MMP-9) and cytokine cascades (tumor growth factor *β*1) as well as from other senescence-related pathways [135–137]. Metalloproteinases' imbalance then results in smooth muscle cells' migration, arterial remodelling, and formation of atherosclerotic lesions [135–137].

This stiffening of the extracellular matrix contributes to the disruption of normal cell-to-cell junctions within the endothelial monolayer. As a result, endothelial permeability is increased, allowing lipoproteins and immune cells to infiltrate the intima. These inflammatory components in combination with endothelial cellular debris initiate the formation of an atherosclerotic lesion, establishing the starting point of atheromatous disease [27, 28, 74, 81].

## 4. Conclusions and Perspectives

Carotid atheromatous disease and stenosis is a major factor of mortality among the elderly [3, 20, 138]. Advancing age is linearly associated with arterial stiffness and strongly associated with thickening of the vascular wall as assessed with cIMT and pulse wave velocity [21–25]. Alterations involving CA diameter and tortuosity increase along with age, while histological findings reveal consistent structural deformation [21–26, 139, 140].Moreover, atheromatous plaques develop with their score increasing along age [25]. The aforementioned changes are explained by various processes at the cellular level including loss of elastic vessel wall components, chronic inflammation, oxidative stress, senescence, and loss of cellular homeostasis. Multiple regulatory molecules are implicated interacting with each other [25, 27, 138, 140]. Moreover, coexistence of aging with other risk factors, such as DM, has cumulative deteriorating effects on the vascular wall [141–145]. Elucidation of aging-induced pathways has expanded our perspective on the relationship between aging and CD [141–143]. The immersion of the field of senescence has also added valuable knowledge towards a deeper understanding of the pathogenic mechanisms [146–149]. Deciphering “vascular senescence” has led to the identification of intracellular targets for the development of novel therapies and new prevention and diagnostic strategies [150–154].

## Supplementary Materials

Supplementary Materials This includes a figure summarizing vascular ageing mechanisms and procedures that are described in the manuscript.
