# Supplementary material for: Carotid Disease and Ageing: A Literature Review on the Pathogenesis of Vascular Senescence in Older Subjects
Source: Curr Gerontol Geriatr Res. 2020 Jun 13;2020:8601762. doi: 10.1155/2020/8601762 (PMC7306882; doi:10.1155/2020/8601762)
Supplement: Supplementary Materials — This includes a figure summarizing vascular ageing mechanisms and procedures that are described in the manuscript. [file 8601762.f1.docx]

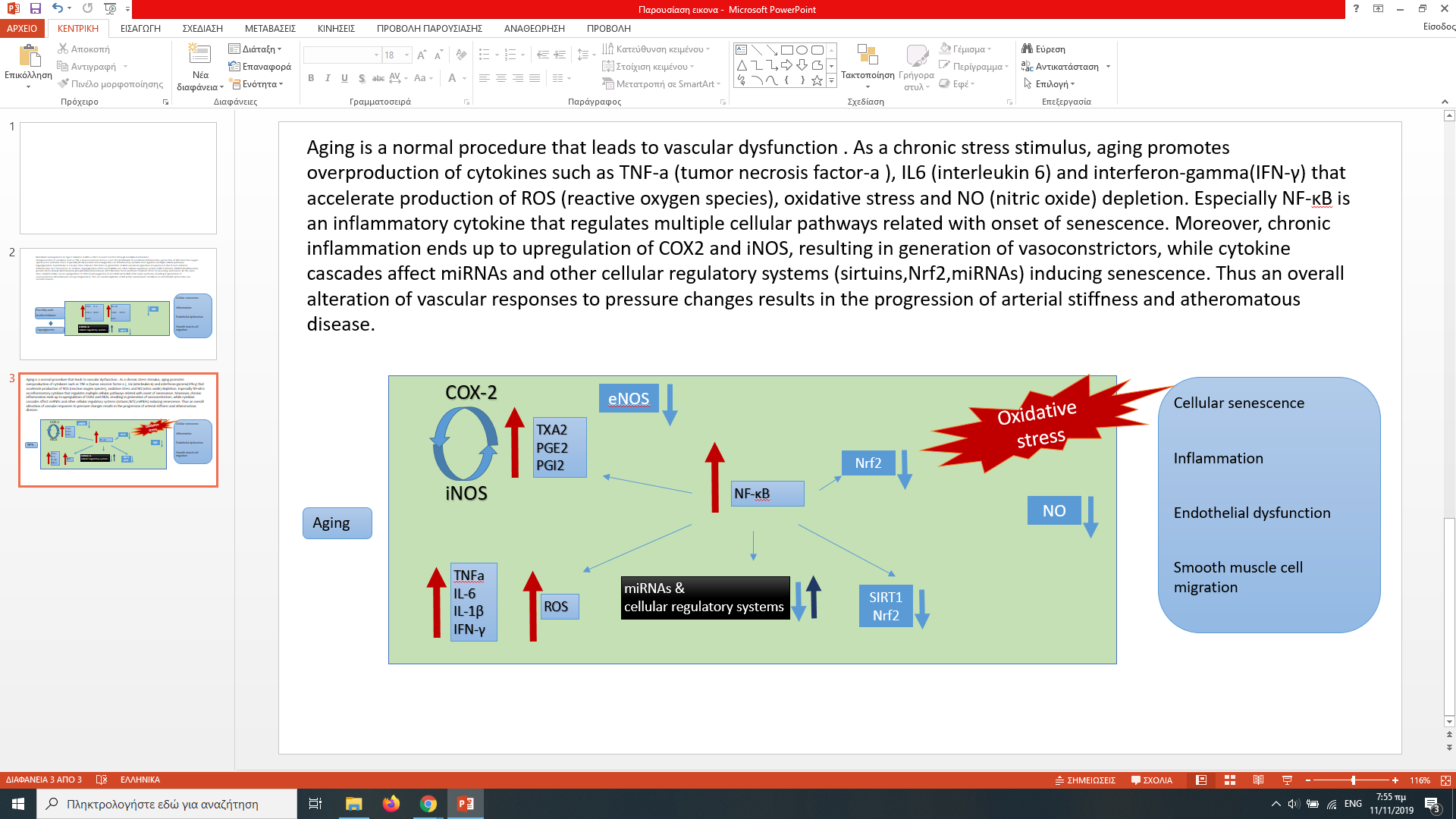


Aging is a normal procedure that leads to vascular dysfunction. As a chronic stress stimulus, aging promotes overproduction of cytokines such as TNF-a (tumor necrosis factor-a), IL6 (interleukin 6) and interferon-gamma(IFN-γ) that accelerate production of ROS (reactive oxygen species), oxidative stress and NO (nitric oxide) depletion. Especially NF-κΒ is an inflammatory cytokine that regulates multiple cellular pathways related with onset of senescence. Moreover, chronic inflammation ends up to upregulation of COX2 (cyclooxygenase 2) and iNOS (inducible NO synthase), resulting in generation of vasoconstrictors, while cytokine cascades affect miRNAs and other cellular regulatory systems (sirtuins,Nrf2, miRNAs) inducing senescence. Thus an overall alteration of vascular responses to pressure changes results in the progression of arterial stiffness and atheromatous disease.
